# Return to Athletic Activity of a Shetland Pony Mare with Coxofemoral Luxation Treated by Femoral Head Ostectomy

**DOI:** 10.3390/ani15040497

**Published:** 2025-02-10

**Authors:** Liliana Carnevale, Tania Tagliabue, Vanessa Rabbogliatti, Roberto Bona, Francesca Cavallier

**Affiliations:** 1 Department of Veterinary Medicine and Animal Sciences, University of Milan, 26900 Lodi, Italy; vanessa.rabbogliatti@unimi.it (V.R.); francesca.cavallier@hotmail.com (F.C.); 2 Independent Researcher, 20100 Milano, Italy; taniatag@hotmail.com; 3 Independent Researcher, 10100 Torino, Italy; robibonavet@libero.it

**Keywords:** horse, hip joint, coxofemoral luxation, ostectomy, ultrasonography, Shetland pony

## Abstract

Background: Femoral head ostectomy is considered a salvage procedure in the treatment of chronic coxofemoral joint luxation in small equids. Permanent lameness of varying degrees, largely depending on the animal’s weight, and potential contralateral limb deformity may occur. The purpose of this study was to report the outcome of a small equid that, despite undergoing a femoral head ostectomy for a chronic coxofemoral joint luxation, was able to return to being ridden by children and to athletic activities. Methods: An 8-year-old Shetland pony mare (180 kg) was presented for a chronic right hind limb lameness (5/5 AAEP grading score) of 12-days duration caused by a craniodorsal coxofemoral joint luxation and permanent upward fixation of the right patella. A femoral head and neck ostectomy was performed concomitant with desmotomy of the medial patellar ligament. Results: Eighteen months after surgery, the mare began being ridden by children and was used for schooling purposes. Five years post-surgery, she continued her work activities with no apparent signs of lameness or discomfort. Conclusions: Femoral head ostectomy following chronic coxofemoral joint luxation can result in a favorable outcome, with equids weighing up to 180 kg potentially regaining soundness and resuming athletic activities.

## Simple Summary

Coxofemoral joint luxation is an uncommon condition in horses but occurs more frequently in ponies, miniature horses, and foals. The primary cause is generally trauma. Upward fixation of the patella frequently accompanies coxofemoral joint luxation in ponies and can present either as a primary condition or as a secondary consequence of the luxation. Clinical signs include varying degrees of lameness, limb shortening, and outward rotation of the stifle and toe. Two treatment options are available: closed reduction and open reduction. The first one is recommended only for cases with a duration of up to 36 h. In the case of reluxation or in chronic cases, open reduction combined with a joint stabilization technique provides a higher likelihood of success. Femoral head ostectomy is regarded as a salvage procedure and is performed when neither closed nor open reduction is feasible. Horses treated with a femoral head ostectomy are typically designated for pasture, for breeding, or as pets. In the present case, we treated a chronic coxofemoral joint luxation, with concomitant permanent upward fixation of the patella, with a femoral head ostectomy without performing a greater trochanter osteotomy. The successful outcome, with a return to athletic activities despite the guarded prognosis, is confirmed through a 5-year follow-up.

## 1. Introduction

Luxation of the coxofemoral joint is a rare condition in equids [1,2,3,4,5]. This condition is described more commonly in young horses, ponies, and miniature horses [3,4,6]. This issue has also been reported in calves, donkeys, Quarter Horses, and Arabian horses [7,8,9,10]. The rarity of this condition in equids is attributed to the deep acetabulum, reinforced by the fibrocartilaginous acetabular rim, robust ligamentous structures (including the round and accessory ligaments), and substantial musculature that provides strong stabilization to the coxofemoral joint [2,10]. The causes of coxofemoral joint luxation include trauma such as falls [2,10], kicks from other animals [2,11], limb entrapment [8], hind limb casting [12], and complications during anesthetic recovery [13]. Several reports have described upward fixation of the patella in conjunction with coxofemoral joint luxation, occurring either as an intermittent pre-existing issue or as a persistent locking coinciding with the acute onset of the luxation [10,11,14,15]. Treatment options include closed-reduction for recent cases presented within 36 h [16]. If closed-reduction is unsuccessful or in more chronic cases, open-reduction and stabilization techniques are recommended. When open-reduction is not feasible, femoral head ostectomy remains a viable salvage procedure. Animals treated this way are intended to be kept as companion animals, for breeding or at pasture [1,3,5,17,18,19]. The aim of this case report is to detail the clinical findings, surgical technique, and long-term follow-up of a Shetland pony mare treated with femoral head ostectomy for craniodorsal coxofemoral joint luxation, resulting in a return to athletic activity.

## 2. Materials and Methods

### 2.1. Case History

**Figure 1:**
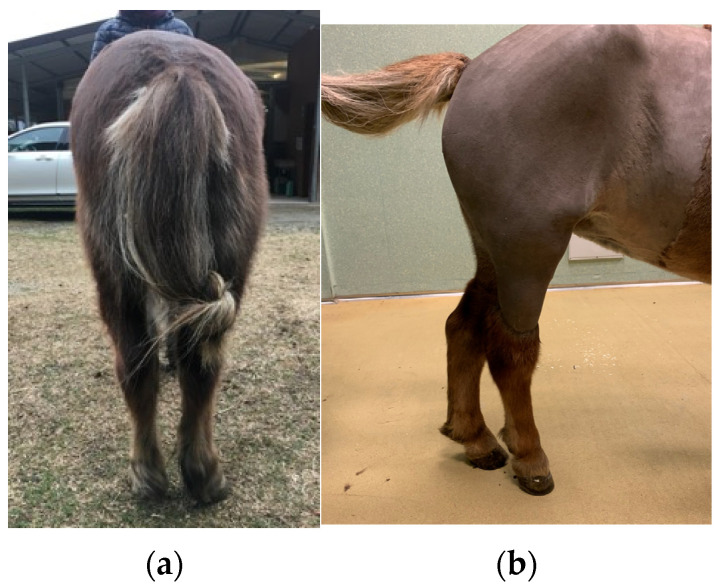
Caudal (**a**) and lateral aspect (**b**) of the right hind limb of the pony mare with atrophy of the quadriceps femoris, biceps femoris, and middle gluteal muscles and outward rotation of the limb.

An 8-year-old Shetland pony mare (180 kg) was presented with a non-weight bearing lameness (5/5 American Association of Equine Practitioners grading score) on the right hind limb (RH) while she was toe-touching at rest. The mare, prior to the lameness, was used for riding school and mounted games. The owner reported severe lameness on the right hind limb of 4-months duration and intermittent upward fixation of the right patella. Worsening of the lameness was observed over a period of 12 days, until the mare could no longer move around in the stable and was unable to lie down The lameness had not responded to stall rest. At presentation, the pony was in good physical condition and all vital parameters where within normal limits. The mare presented a right hind limb non-weight bearing lameness with the leg locked in extension because of a permanent upward fixation of the right patella. There was also outward rotation of the right hind limb with concomitant severe atrophy of the middle gluteal and quadriceps femoris muscles, palpable asymmetry of the greater trochanter of each femur, with the right being higher than the left. The right hind-limb looked shorter due to the tuber calcanei being positioned more proximally compared to the left side (Figure 1). A presumptive diagnosis of right coxofemoral joint luxation was made.

### 2.2. Diagnostic Imaging

**Figure 2:**
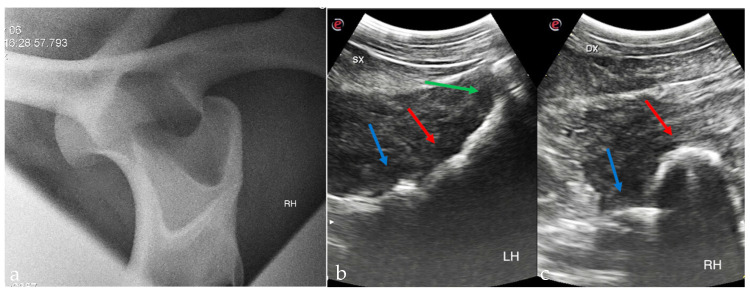
Standing medial 30° cranial 20° dorsal-lateral caudal oblique radiographic view of the right coxofemoral joint (**a**) showing dislocation of the femoral head cranial to the acetabulum (cranial is to the left); ultrasonography of the normal left coxofemoral joint (**b**), green arrow—greater trochanter, red arrow—femoral neck, blue arrow—acetabulum; ultrasonography of the right coxofemoral joint (**c**) with the femoral head (red arrow) dorsally dislocated from the acetabulum (blue arrow).

Standing radiography was performed (Gierth 80 kv 20, Sound Eklin) and a medial 30° cranial 20° dorsal-lateral caudal oblique view of the right coxofemoral joint confirmed the craniodorsal luxation of the femoral head (Figure 2). Mild remodeling of the cranial aspect of the acetabulum was also observed. Additionally, there were small periarticular osteophytes in the distal medial aspect of the femur and the proximal medial aspect of the tibia. On ultrasonographic investigation (Esaote Mylab Gamma) the right femoral head appeared as a curved echogenic line cranio-dorsally displaced relative to the acetabulum (Figure 2).

### 2.3. Surgery

**Figure 3:**
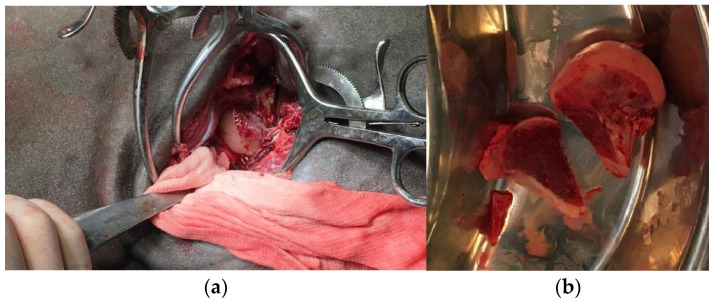
Intra-operative aspect of the luxated femoral head (**a**); femoral head and neck removed with a double cut (**b**).

Food was withheld for 12 h before general anesthesia, whereas water was offered ad libitum. A 14-gauge × 64 mm (Surflo^®^, Terumo Europe N.V., Leuven, Belgium) catheter was then placed into the right jugular vein following skin clipping, disinfection, and desensitization of the insertion site with 1 mL of Lidocaine 2% (Lidocaina 2%, ECUPHAR ITALIA S.r.l, Milano, Italy). The mare was sedated with intravenous (IV) acepromazine (Prequillan 1%, FATRO S.p.A., Bologna, Italy) at 0.03 mg/kg, and then the mouth was rinsed. Fifteen minutes following acepromazine administration, the pony was sedated with detomidine (Domosedan, Orion Pharma S.r.l., Milano, Italy) at 10 μg/kg IV. Anesthesia was induced with IV diazepam (Ziapam, Laboratoire TVM, Mougins, France) at 0.05 mg/kg and ketamine (Ketavet 100, MSD Animal Health S.r.l., Milano, Italy) at 2.5 mg/kg. Once the horse was in lateral recumbency, orotracheal intubation was performed (silicon tube, internal diameter 18 mm). Anesthesia was maintained with isofluorane delivered in a mixture of oxygen (O_2_) and air, so as to maintain the inspired O_2_ fraction (FiO_2_) between 60 and 65%. While the mare was anesthetized, we successfully released the right stifle joint from the permanent upward fixation of the patella with the assistance of a chain-hoist. Subsequently, closed-reduction of the right coxofemoral joint luxation was attempted unsuccessfully for 30 min using the same chain-hoist to apply traction to the affected limb combined with outward rotation and adduction. At this stage, the decision to perform open surgical reduction was taken. The mare was positioned onto left lateral recumbency. The surgical area was clipped and cleaned for aseptic surgery with 2% chlorhexidine gluconate scrub (Clorexinal 2%, Nuova Farmec, Verona, Italy). A craniolateral approach to the right coxofemoral joint was performed [20]. A 15 cm straight skin incision centered on the coxofemoral joint and parallel to the biceps femoris muscle was made. After dissection of the fascia lata, the biceps femoris was separated from the superficial gluteal muscle and the craniodorsal aspect of the greater trochanter was identified. The middle gluteal muscle was retracted dorsally while the insertion of the vastus lateralis was partially reflected caudally to allow exposure of the femoral neck. The femoral head was palpated craniodorsal to the acetabulum. Three Gelpi retractors were inserted to improve exposure of the surgical area. With the limb externally rotated, a Homhann retractor was carefully inserted beneath the luxated femoral head to improve mobilization and exposure. Severe damage of the cartilage with exposure of the subcondral bone of the femoral head was evident. The joint luxation could not be reduced because of its duration and muscle contraction. Femoral head and neck ostectomy was considered to be the best option for this pony. A first cut was performed with an oscillating bone saw (De Soutter Medical, Aston Clinton, United Kingdom) and the head of the femur with the proximal part of the neck was removed, thus improving access to the base of the neck itself. A second cut parallel to the previous one was performed with the same surgical device at the base of the femoral neck, as close as possible to the femoral shaft (Figure 3). Further neck debridement was achieved with a rongeur and an electric burr (De Soutter Medical, Aston Clinton, UK). The surgical site was rinsed with Lactated Ringer’s Solution (Galenica Senese, Siena, Italy) throughout the entire surgical procedure. A fat-pad graft (~5 × 5 cm) was harvested from the precrural region and placed between the femoral ostectomy site and the acetabulum as an attempt to prevent rubbing of the femur on the acetabular rim. The graft was sutured in a simple interrupted pattern (USP 2-0 polydioxanone, Ethicon^®^ LLC, Roma, Italy) to the deep gluteal muscle and the remnants of the joint capsule. Wound closure involved apposition of the deep gluteal and superficial gluteal muscles in a continuous chained suture (USP 2-0 polydioxanone, Ethicon^®^ LLC, Roma, Italy). The cut surface of the fascia lata was secured to the biceps femoris muscle in simple continuous fashion (USP 2-0 polydioxanone, Ethicon^®^ LLC, Roma, Italy). The skin was closed in a simple interrupted mattress suture (USP 2-0 polypropylene, Ethicon^®^ LLC, Roma, Italy) and a stent bandage was sutured over the wound. A desmotomy of the medial patellar ligament of the right stifle was subsequently performed through an ~2 cm skin incision centered on the distal aspect of the ligament. Skin closure was performed in a simple mattress-interrupted fashion (USP 2-0 polypropylene, Ethicon^®^ LLC, Roma, Italy). This procedure was carried out to prevent the recurrence of upward fixation of the patella [10,14,15,21]. At the end of the surgery, the pony was kept under general anesthesia until she was transported into the box using a fork-lift. The recovery from anesthesia was uneventful and assisted by the anesthetist with head and tail manually supported.

### 2.4. Post-Operative Treatment

Following surgery, the mare received Ceftiofur (2.2 mg/kg IV BID) (Wondercef, Fatro S.p.A., Bologna, Italy) for 7 days, Meloxicam (0.8 mg/kg IV BID) (Meloxidolor, Dechra Veterinary Products S.r.l, Torino, Italy) for 10 days, then Meloxicam (0.6 mg/kg PO SID) for another 20 days and Sucralfate (30 mg/kg PO TID) (Sucralfin 2 gr., Sanofi, Milano, Italy) for 20 days.

The stent bandage was removed after 3 days. The surgical incision was cleaned daily with a povidone-iodine solution (Pharmac s.r.l., Rozzano, Italy), followed by the application of gentamicin ointment (Gentalyn 0,1%, Essex, Milano, Italy]. Through the immediate post-surgical period, the mare was forced to exercise daily by hand-walking for five minutes. Starting the day after the surgical procedure, an electromechanical massager (Niagara Equissage Pulse^®^ Digital, Equissage, Newmarket, ON, Canada) was applied locally to the quadriceps femoris and gluteal muscles. This treatment was administered twice daily for a duration of 15 min over a period of 20 days. Two days post-surgery, the mare showed an increased ability to bear weight on the affected limb while standing and was able to take a few steps, exhibiting a right hind limb lameness (RH 4/5 AAEP) at walk. Additionally, the mare partially lost the habit of bringing the limb forward and keeping it adducted at rest, as it was before the surgery. Five days after surgery, the mare was forced to exercise by hand-walking twice daily for 5 min. The pony was still lame, but the lameness was improving (RH 3/5 lameness at walk). The cranial phase of the step was still quite short but better compared with the immediate post-surgery period (Video S1).

The sutures were removed 12 days after surgery. Minimal swelling and no discharge were present at this stage. The surgical incision healed by first intention. Mild improvement of the gluteal and quadriceps femoris muscles tone of the RH limb was noted. The mare was more willing to walk and mild to good improvement of the lameness was evident (RH 2/5 lameness at walk). Twelve days after surgery, the pony began to lie down and get up on her own. When the mare was discharged from the hospital, 21 days after surgery, her gait was much better and the cranial phase of the step was further improved. Instructions to the owner were to continue with the box-rest and hand-walking exercises, progressively increasing for up to 30 min three times a day.

### 2.5. Follow-Up

**Figure 4:**
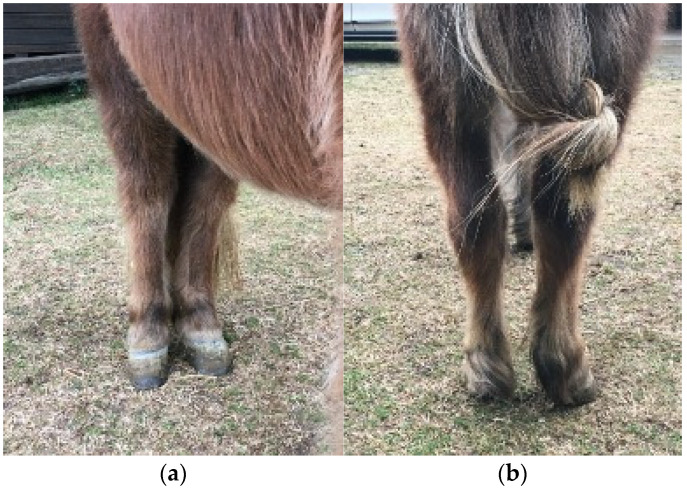
Clinical appearance 1-year post surgery. Both hind limbs exhibit equal weight bearing Front view (**a**) and rear view (**b**).

One month after surgery, the mare was re-evaluated and standing X-rays were taken. The pony was still lame (RH 2/5 lameness at walk) and shortening of the cranial phase of the step was visible. The radiographs revealed small periarticular osteophytes at the level of the right acetabulum, and the femur was aligned with the acetabular cavity. The pony continued to undergo regular training at a walk three times a day for half an hour on both hard surfaces and grass. Large circles on both hands were introduced for the next eight weeks. At 3 months after surgery, no improvement of the lameness was present and the mare was showing some pain on the passive flexion of the limb. Despite the right hind limb lameness, the pony was forced to continue physiotherapy at a walk with three daily sessions of 30 min each for the following sixteen weeks. In addition, she also began walking on inclines for 5 min three times a day. At 7 months after surgery, no more pain was elicited at the passive flexion of the limb. The pony started to be turned out in a small paddock and 5 min at trot was introduced in the daily exercise. At walk, only a very marginal deficit in gait was apparent (RH 1/5 lameness at walk), while moderate lameness was present at trot (RH 3/5 lameness at trot). One year after surgery, the mare was sound at walk. Mild atrophy of gluteal and quadriceps femoris muscles was present. Both hind limbs were kept in a completely normal position while standing (no outward rotation of the injured limb) (Figure 4) and no pain was elicited at passive flexion of the operated limb. At this stage, the mare was regularly lunged at trot for 30 min daily and canter was introduced (Video S2). At dynamic examination at trot, 1/5 lameness on straight line on hard surface, 2/5 lameness on right-side circle on soft ground and a slightly more pronounced lameness on left-side circle on soft ground were detected. In canter the mare showed a close spatial and temporal placement of the hind limbs. This was partially attributed to hyper-excitability and high-speed movement while free.

## 3. Results

**Figure 5:**
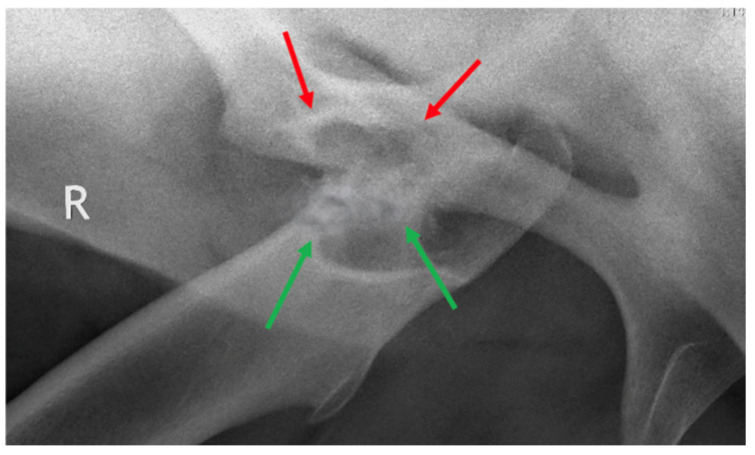
Standing medial 30° cranial 20° dorsal-lateral caudal oblique radiographic view of the right coxofemoral joint, 5 years post-op. A pseudoarthrosis is evident. The acetabulum (red arrows) appears more shallow and smaller than pre-operatively. Large and irregular new bone formation at the femoral ostectomy site (green arrows) is clearly visible. The cranial is to the left.

Eighteen months after surgery, the pony started to be ridden again by young children and went back to light work. Mild lameness was visible at trot only on the left circle (RH 2/5 lameness). No complications were reported in the 24 months following surgery. No apparent signs of residual lameness were observed on video footage sent for update by the owner. Five years after the operation, the right coxofemoral joint was re-evaluated radiographically, revealing a pseudoarthrosis. There was irregular new bone formation at the ostectomy site of the proximal aspect of the femur. The acetabulum appeared more shallow, smaller, and filled with dense bone compared with pre-operatively (Figure 5). Currently, although the mare’s locomotion remains irregular, notably with a lack of right hind limb engagement, her comfort is compatible with schooling use (Video S3).

## 4. Discussion

This case report demonstrates that a chronic coxofemoral joint luxation in a 180 kg Shetland pony can be effectively managed through a femoral head ostectomy without greater trochanter osteotomy, allowing for a successful return to riding and athletic activities. Femoral head ostectomy is generally considered a salvage procedure for small equids and ponies with chronic coxofemoral joint luxation [16,17,22,23]. The prognosis for animals surviving the procedure is favorable if the intended use is for pasture, breeding, or as companion animals. Additionally, patient prognosis after this kind of surgery is mainly dependent on body weight (less than 100 kg) [16,17,24]. Several factors contributed to improving the positive outcome for this case. The proactive physiotherapeutic intervention, started immediately after surgery and continued despite the pony exhibiting lameness in the right hind limb at one- and three-months post-surgery, aimed at promoting the earliest possible formation of pseudoarthrosis. It was crucial to perform two and later three physiotherapy sessions daily, gradually extending the duration of the exercise sessions each week, and to place the pony in the paddock daily seven months post-operatively. The radiographic appearance of the right coxofemoral joint pseudoarthrosis in the long-term follow-up resembles that reported in dogs and in an experimental study on sheep [25,26,27]. However, the relationship between the imaging findings of the ostectomy site in the immediate post-operative period, the subsequent development of pseudoarthrosis, and the long-term clinical outcome remain highly controversial and unproven in small animals [25]. We believe that, in the case described herein, the radiographic findings of the pseudoarthrosis could not be directly associated with the favorable clinical outcome, characterized by a functional, pain-free false joint. The only potentially relevant factor may be the physiological positioning of the right femur, that is not dorsally dislocated, as sometimes occurs [18]. To the authors’ knowledge, there have been 12 equids, including ponies, miniature horses and foals (seven Shetland ponies) weighing between 67 kg and 233 kg at the time of surgery, that have undergone femoral head ostectomy to treat a cranio-dorsal coxofemoral joint luxation [1,4,9,11,14,17,18,24,28]. Two pony Shetland were euthanized during the immediately post-operative period because of complications [17]. Eight ponies or miniature horses were intended for pasture, as pets, or for breeding purposes [1,4,11,14,17,24,28]. A Welsh pony, 4-years-old (233 kg) with a 4-year follow-up, developed a hyperextended fetlock on the contralateral hind limb and a 4/5 lameness on the operated one. Despite his compromised condition in both hind limbs, he was able to trot and canter on his own while at pasture [18]. An Arab filly operated on her right hind limb at 1 month of age (70 kg) was being used for breeding at a 4-year follow-up (400 kg). The mare continued to exhibit lameness on the right hind limb and developed a limb deformity on the counterpart [9]. The case described in this report is the first one featuring a long-term and well-documented follow-up, showing a return to an acceptable activity level, including riding and regular schooling, after excision arthroplasty in a pony weighing 180 kg.

Closed-reduction in coxofemoral joint luxation is more likely to be successful if performed within 36 h after luxation [16,23]. In cases where this is unsuccessful or in chronic cases, several surgical options have been described. These included prosthetic capsule technique, toggle pin fixation, prosthetic capsular reconstruction, and transposition of the greater trochanter of the femur [6,29]. Recently, a modified toggle pin technique, combined with prosthetic capsular reconstruction, was proposed to treat a 2-year-old Shetland pony weighing 167 kg [21]. The outcome indicated that the pony was sound at both walk and trot, however, the specific intended use of the animal was not disclosed. Total coxofemoral joint arthroplasty is considered a viable option only in very small equids [30]. Only one report describes a long-term successful outcome in a miniature horse (85 kg) [31]. Thirty-two months post-surgery, the miniature horse remains sound at both trot and canter at pasture.

The pony described in this case report was presented with concomitant permanent upward fixation of the right patella, resulting in the right hind limb being locked in extension. In the authors’ opinion, the coxofemoral joint luxation was likely secondary to the upward fixation of the patella, as anamnesis indicated that the pony had been lame for approximately 4 months, with episodes of intermittent upward fixation of the patella. Although in some instances the pony was able to self-release the patella, there were other occasions where human intervention was required to facilitate its release. Coxofemoral joint luxation occurs as a result of the violent contraction of the quadriceps femoris muscles during attempts to flex the limb while it is trapped in extension, combined with the inability of the stifle joint to flex when locked [15]. For this reason, the surgical procedure for this mare also included a medial patellar ligament desmotomy to prevent the recurrence of upward fixation of the patella.

Radiographs acquired with the pony standing and ultrasonographic examination can provide an accurate diagnosis of coxofemoral joint luxation, as in this case [11,32,33,34,35,36].

The surgical procedure involved smoothing the ostectomy site using an electric burr unit and rongeurs, as recommended to prevent bony contact with the acetabulum, which could lead to bone fragmentation and associated discomfort [18,37,38]. In small animals, some authors suggest that better outcomes can be achieved by interposing soft tissue, such as muscle, between the femoral neck and the acetabulum [39]. In the present case, we utilized a fat pad graft to help prevent bony contact. While one author (LC) commonly performs this type of procedure in dogs and cats, its effectiveness in this context has not been proven and remains a hopeful approach.

During the immediate post-operative period and until discharge, the mare was treated with an electromechanical massager (Niagara Equissage Pulse^®^ Digital, Equissage, Newmarket, Canada) applied to both the quadriceps femoris and gluteal muscles. This treatment aimed to enhance local blood circulation, improve venous drainage, and relieve muscle tension. The device operates on the principle of Cycloidal Vibration Therapy (CVT), a three-dimensional vibration generated by a sinusoidal electromechanical oscillator [40]. A similar approach using an electrical stimulation unit (TENS) was reported in the post-operative care of a heifer undergoing a femoral head ostectomy as a treatment for coxofemoral joint luxation [41]. Restoring limb function through the formation of a pseudoarthrosis requires starting rehabilitation as early as possible. In this case, the pony began walking on the second day after surgery, initially with hand-walking on a hard surface. The walking exercises were increased gradually, and during the recovery period exercise on grass surfaces was introduced. After returning home, the rehabilitation regimen was progressively intensified, and by 18 months post-surgery, the mare was able to be ridden by young children. Currently, 5 years post-surgery, she is actively being ridden and used for schooling purposes without any apparent discomfort. For this pony, the only feasible treatment for coxofemoral joint luxation was femoral head ostectomy, due to the chronic nature of the lesion and the high body weight, which rendered alternative surgical procedures unsuitable [10,15,16]. Prior to surgery, the owner was informed that the best possible outcome would be to maintain the pony as a pasture animal and companion. The authors of this paper were positively surprised by the outcome, as it was unexpected when compared to reports in the literature [1,4,9,11,14,17,18,24,28]. Additional factors contributed to the favorable outcome in this case: the pony’s active nature and well-developed musculature (she was a pony used in mounted games), her young age (an 8-year-old), the accuracy in performing the femoral neck ostectomy as close as possible to the femoral shaft [37], the absence of post-operative complications in both the short- and long-term, and the meticulous post-operative care and physiotherapy closely supervised by two of the authors (T.T., R.B.) while the mare was at home.

## 5. Conclusions

Femoral head and neck ostectomy following chronic coxofemoral joint luxation can result in a favorable outcome, with equids weighing up to 180 kg potentially regaining soundness and resuming athletic activities. This low-cost procedure represents an effective alternative to more complex techniques that require implants and have uncertain outcomes in most cases. Moreover, there are currently no evidence-based treatment guidelines for managing coxofemoral joint luxation in horses.

## Data Availability

The original contributions presented in the study are included in the article/Supplementary Material. Further inquiries can be directed to the corresponding authors.

## Supplementary Materials

The following supporting information can be downloaded at: https://doi.org/10.5281/zenodo.14498045 (accessed on 4 February 2025). Video S1: Video demonstrating immediate pre-operative lameness and inability to ambulate unassisted, right hind limb locked in extension when the mare was anesthetized, followed by ambulation at 2 days post-surgery and improvement at walking 18-days post-surgery. https://doi.org/10.5281/zenodo.14743780 (accessed on 4 February 2025). Video S2: Video showing the mare at trot on straight line on hard surface, lunged at trot, and canter on her own, one-year post-surgery. https://doi.org/10.5281/zenodo.14577374 (accessed on 4 February 2025). Video S3: Video showing the pony being ridden 18 months after surgery and 5 years post-operatively.
